# The role of simulation in the development of technical competence during surgical training: a literature review

**DOI:** 10.5116/ijme.513b.2df7

**Published:** 2013-03-16

**Authors:** Matthew P. Thomas

**Affiliations:** Department of Vascular Surgery, James Cook University Hospital, Marton Road, Middlesbrough, United Kingdom

**Keywords:** Simulation, surgical training, virtual reality, box trainers, robotics

## Abstract

**Objectives:**

To establish the current state of knowledge on the effect of surgical simulation on the development of technical competence during surgical training.

**Methods:**

Using a defined search strategy, the medical and educational literature was searched to identify empirical research that uses simulation as an educational intervention with surgical trainees. Included studies were analysed according to guidelines adapted from a Best Evidence in Medical Education review.

**Results:**

A total of 32 studies were analysed, across 5 main categories of surgical simulation technique - use of bench models and box trainers (9 studies); Virtual Reality (14 studies); human cadavers (4 studies); animal models (2 studies) and robotics (3 studies). An improvement in technical skill was seen within the simulated environment across all five categories. This improvement was seen to transfer to the real patient in the operating room in all categories except the use of animals.

**Conclusions:**

Based on current evidence, surgical trainees should be confident in the effects of using simulation, and should have access to formal, structured simulation as part of their training. Surgical simulation should incorporate the use of bench models and box trainers, with the use of Virtual Reality where resources allow. Alternatives to cadaveric and animal models should be considered due to the ethical and moral issues surrounding their use, and due to their equivalency with other simulation techniques. However, any use of surgical simulation must be tailored to the individual needs of trainees, and should be accompanied by feedback from expert tutors.

## Introduction

Surgical training has traditionally been modelled on an apprenticeship system, where trainees learn by direct instruction from their seniors, combined with long-term observation and assessment from those same seniors. This is accompanied by “the gradual absorption in to a ‘community of practice’ [where] participants learn as much from their peers”.^1^ However, this traditional model has seen significant changes in recent years, driven by the European Working Time Directive (EWTD), a piece of European-wide legislation that as of August 1st 2009, introduced a maximum 48-hour working week for most doctors-in-training, including trainee surgeons.^2^It has been estimated that consultant surgeons have previously reached their high level of expertise after 30,000 hours of “on-the-job” training, gained through the traditional apprenticeship approach; post-EWTD, this has been revised to just 6000 hours.^3^ Such a reduction in hours has led to an obvious decrease in training opportunities in the operating theatre – in one region of the UK, it has been estimated that training operations have been reduced to less than a third of the minimum recommended number, and that providing trainees with the requisite training operations would require an extra 270 theatre days a year at a cost of £1.3 million.^4^ Such changes to working hours are therefore of significant concern to those responsible for the training of surgeons, and to trainees themselves, and have led to the search for effective methods of increasing technical skills that can be delivered outside of the operating theatre. Surgical simulation is one such method. Simulation describes “the technique of imitating the behaviour of some situation or process…..by means of a suitably analogous situation or apparatus”.^5^ In addition to the EWTD, there are also other significant drivers to the use of simulation in surgical training. Concerns about patient safety and the need to significantly reduce avoidable medical errors have created what has been called by some an “ethical imperative” for the use of simulation in medical education, where “patients are to be protected whenever possible……they are not commodities to be used as conveniences of training”.^6^ Simulation away from patients and the clinical environment allows technical procedures to be broken down in to smaller, component parts that can be practiced repeatedly. This can be done at the trainees own pace, where instant feedback can be provided, both self-feedback and from senior experts. Surgical simulation can also provide training opportunities that are immediate, without having to wait for a particular “real-life” case or pathology to present itself. In a field where technological developments are so rapid, simulation also allows exposure to these new technologies and techniques early in the training period. There is also a cost implication to the use of simulation. With the use of simulation outside of the operating theatre environment, one study suggests a possible saving of $160,000 US during training, due to faster completion of tasks, fewer errors and reduced equipment spoilage costs, plus savings in instructor/teacher costs.^7^ Such considerations have led to the proposal in the US of the need for a national consortium to promote the development of a national simulation system for residency training.^8^There are several different surgical simulation techniques in widespread use, ranging from low-fidelity simple synthetic jigs and box-trainers to higher fidelity animal and cadaver models, through to advanced virtual reality technology. Fidelity within simulation refers to its “exactness of duplication;” in other words, the level of “realism” the simulation technique achieves. High fidelity simulation immerses the user in a more realistic and interactive environment, whereas low fidelity models use materials and equipment that are less similar to those that are actually encountered in the true operating room.^9^ The importance of simulation fidelity in the transfer of learning has been addressed in a recent review. High fidelity simulation was found to show clear gains in performance and transfer to the real patient setting, when compared with “typical opportunistic instruction”. However, when compared to low fidelity simulation, these gains were “more modest” and in most studies, did not achieve statistically significance. It should be noted that this review did not exclusively examine simulation in surgical training, but also evaluated simulation in auscultation skills and in complex crisis management skills as well.^10^The use of simulation itself is not without its disadvantages. If skills learnt during simulation are not practiced regularly thereafter, they may be rapidly lost, but without the practitioner being aware that such a loss has taken place.^1^ Simulation may also become “an end in itself, disconnected from the professional practice for which it purports to be a preparation”.^1^ The repeated practice on “inanimate” simulation devices can also remove the human interaction that is so important in clinical practice.^11^Ultimately, the role of the surgeon is to be able to safely and effectively perform operative techniques and procedures on actual patients, and with the introduction of the EWTD it is the opportunity to practice these techniques in the operating theatre that is directly affected. Both trainees, and those that train them, therefore need to be assured that the use of surgical simulation outside of the operating theatre is a valuable training tool in increasing and developing these technical skills.Therefore, in light of its already extensive use within surgical training, both actual and proposed, and with so many professional bodies advocating its continued widespread use, this review will seek to explore the literature to ask “does the use of surgical simulation make a measurable impact on the development of technical competence in surgical training?” The objectives are:

- To synthesise the current literature on the effectiveness of these techniques in developing and improving  technical competence:
  - Within the simulated environment
  - On transfer to the real surgical patient
- To make recommendations to clinical educators on the effective use of simulation in surgical training, based on this synthesis

## Methods

This research project was conducted as a literature review; formal ethical approval was therefore not required. The following databases were searched, chosen to provide the broadest range of research within the fields of healthcare and educational research: British Nursing Index (1985 to date); CINAHL (1981 to date); EMBASE (1980 to date); Medline (1950 to date); The Cochrane Library (consisting of the Cochrane Database of Systematic Reviews, Cochrane Central Register of Controlled Trials (CENTRAL), Cochrane Methodology Register, Database of Abstracts of Reviews of Effects (DARE), Health Technology Assessment Database and the NHS Economic Evaluation Database); HMIC (covering the Department of Health Library & Information Service, 1983 to date, and the Kings Fund Information & Library Service, 1979 to date); BREI (1975 to date); EPPI-Centre Database of Educational Research; CERUK (2000 to date); OpenGrey database (1980 to date) and the Conference Papers Index (1982 to date).These databases were searched using the following keywords and Boolean combinations: surgical AND training; surg* AND simulat* (use of wildcard * symbol to find surgery/surgical, and simulation/simulator); technical AND skills; technical AND competence; “virtual reality” (use of “ ” to find exact term); cadaver* (use of wildcard * symbol to find cadaver/cadaveric); robot* AND simulat* (use of wildcard * symbol to find robot/robots/robotic, and simulation/simulator); animal AND simulat* (use of wildcard * symbol to find simulation/simulator). The following thesaurus terms for the above keywords were also used: “computer simulation”; “computer-assisted instruction”; “patient simulation”.The search was conducted looking for English language articles, and the title and abstract of articles identified by the above strategy were then screened. Articles were finally included where they described empirical, comparative research which utilised simulation techniques as an educational intervention. In order to keep the review as broad and extensive as possible, studies were included from any of the 9 recognised major surgical specialties (Cardiothoracic Surgery, General Surgery, Neurosurgery, Oral and Maxillofacial surgery (OMFS), Otolaryngology (ENT), Paediatric surgery, Plastic surgery, Trauma and Orthopaedic Surgery (T&O) and Urology), and also studies involving Obstetrics & Gynaecology trainees, a specialty which also necessitates training in surgical skills. “Citation tracking” was used to review the reference lists of all those articles meeting these criteria, in order to identify further potentially relevant work.The focus of the review is the use of simulation in surgical training. Surgical training itself begins once medical school is completed, after a certain generic level of basic clinical competence has already been reached. Therefore all literature related to the use of surgical simulation techniques with those surgical trainees who have chosen to embark on a surgical career and started their surgical training has been included in the review. Literature focussing solely on the use of surgical simulation techniques in medical students was excluded. For the same reasons, the use of simulation solely in those who have completed their surgical training (e.g. the “expert surgeon”, such as surgical consultants) was also excluded. Studies comparing the experience of surgical trainees with medical students and/or “expert” surgeons have been included where they presented data on the surgical trainees as a separate participating group. In order to determine this, studies must have clearly defined the level of experience/training of the participants; where no clear definition of this level was found, the study was excluded.All studies meeting the final inclusion criteria were initially classified by the type of surgical simulation technique used, in order to categorise the available types of surgical simulation and to allow studies detailing similar surgical simulation techniques to be grouped together. Once grouped, studies were then further analysed following a system based on guidelines for educational studies involving simulators produced by the BEME Collaboration.^12^ Data was collected from this analysis on a data extraction sheet. The assessment of study design for each study was then summarised using the data extraction sheets, under the following headings: study authors/year; methodology; participants; intervention (simulation task used); outcome measures; method of evaluation (including pre-intervention measurement); results and evidence of transfer to clinical environment. A single author (MT) was responsible for the initial literature search, the screening of article titles and abstracts and the decision to finally include each article. The same author was also solely responsible for the data extraction and analysis.

## Results

A total of 74 studies were identified using surgical simulation as an educational intervention. When full inclusion and exclusion criteria were applied, a final total of 32 studies qualified for full analysis. These studies fell across five main categories of surgical simulation technique - use of bench models and box trainers; Virtual Reality; animal models; human cadavers and robotics. The main study characteristics are summarised in Table 1.

**Table 1:** Summary of study characteristics for included studies describing empirical research involving simulation in surgical trainees

Study		Methodology		Participants		Intervention (simulation task)		Outcome measures		Method of evaluation		Results	Evidence of transfer to clinical environment?	
Derossis *et al*, 1998 ^13^		Randomised control trial; prospective		12 Surgical residents, all PGY3^*^		5 weekly practice sessions on inanimate box trainer vs no simulation practice		Task completion time; “precision of performance” score; overall score based on the above		Evaluation on 7 tasks on box trainer, before and after intervention		Significant improvement for all 7 tasks and total score practice group; significant improvement 4 out of 7 tasks and total score no practice group; improvement significantly greater in practice group	No	
Scott *et al*, 2000 ^14^		Single-blind, randomised control trial; prospective		27 Surgical residents enrolled, 22 completed, all PGY2 & 3		Participation in training curriculum on box trainer vs no simulation training		Task completion time; global assessment score covering 8 components of technical procedure		Evaluation on 5 tasks on box trainer and performance of operative procedure on real patient, before and after intervention		Significantly greater reduction in task completion time in training group; significantly greater improvement in 4 out of 8 aspects global assessment score in training group	Yes	
Hamilton *et al*, 2001 ^15^		Single-blind, randomised control trial; prospective		21 Surgical residents, all PGY3 & 4		Participation in training curriculum on box trainer vs. no simulation training		Global assessment score covering 8 components of technical procedure, including composite score		Performance of operative procedure on real patient in operating room, before and after intervention		Significantly higher composite score in 5 out of 8 components of global assessment, plus significant improvement in composite score and in 4 out of 8 components of global assessment in training group	Yes	
Risucci *et al*, 2001**^16^**		Randomised control trial; prospective		14 Surgical residents, all PGY1		2 tasks on inanimate bench model repeated 9 times, either with or without additional instruction (demonstration video and direct feedback)		Task completion time; total number of errors		Pre-test evaluation after completing each task; post-test mean task completion and total error score		Task completion time significantly reduced *both* groups; error score and variance in number of errors significantly greater without instruction	No	
Traxer *et al*, 2001 ^17^		Single-blind, randomised control trial; prospective		12 Urology residents, all PGY3 to 5		10 days skills training on inanimate box trainer vs no skills training		Task completion time; performance score assessing 7 components of technical procedure		Cumulative task completion time on 5 tasks performed on box trainer; performance score on operative procedure performed on live animal		Significant decrease in task completion time in skills training group; performance score significantly improved in *both* groups	No	
Korndorffer *et al*, 2005 ^18^		Single-blind, randomised control trial; prospective		17 Surgical residents, PGY1 to 5		8 weeks practice on inanimate box trainer vs no simulation practice		Task completion time; accuracy errors; knot security; overall score based on all the above		Evaluation of suturing task performed on live animal, before and after intervention		Significant improvement in task completion time, accuracy errors and overall score, plus significantly higher completion time and overall score in practice group; significant improvement in completion time and overall score in no practice group	Yes	
Banks *et al*, 2007 ^19^		Single-blind, randomised control trial; prospective		20 Obstetrics and Gynaecology Residents, all PGY1		Surgical teaching in operating room vs teaching in operating room plus participation in surgical skills laboratory		Written assessment; task-specific checklist; global rating scale; “pass-fail” rating		Evaluation of simulated procedure on inanimate box trainer plus assessment of operative procedure on real patient, before and after intervention		Significantly higher score in all assessments post-intervention in the skills laboratory group	Yes	
Joyce *et al*, 2010 ^20^		Cohort study; prospective; no evidence of randomisation		11 Surgical residents (2 PGY1, 2 PGY2, 2 PGY3; 2 T1^†^, 2 T2, 1 T3)		2 weeks practice on inanimate bench model		Task completion time; Objective Structured Assessment of Technical Skill (OSAT), covering 11 technical components of procedure		Performance of procedure on *ex vivo* animal model, before and after intervention		Significant improvement in task completion time and all 11 component of OSAT	No	
Price *et al*, 2011 ^21^		Single-blind, randomised control trial; prospective		39 Surgical residents (29 PGY1, 10 PGY2)		Expert tutorial vs expert tutorial plus 2 weeks training on bench model		Task completion time; OSAT; global rating scale		Pre-test score on bench model; post-test on live animal model		All outcome measures significantly higher in training group	No	
							
Study	**Methodology**		**Participants**		**Intervention (simulation task)**		**Outcome measures**		**Method of evaluation**		**Results**			**Evidence of transfer to clinical environment?**
Hamilton *et al*, 2002 ^22^	Single-blind, randomised control trial; prospective		50 Surgical residents enrolled, 49 completed (30 PGY1, 19 PGY2)		Training on VR simulator vs training on inanimate box trainer		Task performance score; global assessment score		Evaluation of 6 (VR group) or 5 (box trainer group) tasks, before and after intervention; PGY2 Residents evaluated on procedure on real patient, before and after intervention		Task performance score significantly improved in both groups; global assessment score on real patient significantly improved in VR group only			Yes
Seymour *et al*, 2002 ^23^	Double-blind, randomised control trial; prospective		16 Surgical Residents (PGY1 to 4)		“Standard” training plus training on VR simulator vs “standard” training alone		Task completion time; total number of errors		Evaluation of operative procedure on real patient, after intervention		Statistically significant improvement in error number with less variability in performance in VR group			Yes
Grantcharov *et al*, 2004 ^24^	Single-blind, randomised control trial; prospective		20 Surgical Trainees (median time from graduation 7 years)		Training on VR simulator vs no simulation training		Task completion time; error score; “economy of movement” score		Evaluation of operative procedure on real patient, before and after intervention		Significant improvement in all outcome measures in VR training group			Yes
Dayal *et al*, 2004 ^25^	Cohort study; prospective; no evidence of randomisation		16 Surgical Residents (“Novice” group) + 5 “Expert” Surgeons		2 hours of training on VR simulator, training given by “expert”		Task-specific checklist; “fluoroscopy time”; amount of dye used; subjective evaluation of technical ability		Evaluation of simulated operative procedure on VR simulator, before and after intervention		Significant improvement in all outcome measures in Novice group			No
McClusky *et al*, 2004 ^26^	Double-blind, randomised control trial; prospective^‡^		12 Surgical Residents (PGY 1 & 2)		Training on VR simulator vs “standard” training		Task completion time; error score		Evaluation of operative procedure on real patient, after intervention		Improvement in task completion time and error score in VR group, no evidence of significance			Yes
Andreatta *et al*, 2006 ^27^	Double-blind, randomised control trial; prospective		21 Surgical Interns enrolled, 19 completed		Training on VR simulator vs no simulation training		“Time and accuracy” assessment; global assessment score		Evaluation of operative procedure performed on anaesthetised pig, after intervention		VR group performed significantly better in 5 out of 6 parameters of global assessment score			No
Chaer *et al*, 2006 ^28^	Single-blind, randomised control trial; prospective		20 Surgical Residents		Didactic training plus training on VR simulator vs didactic training alone		Task specific checklist; global assessment score covering 12 components of technical procedure		Evaluation on 2 consecutive technical procedures performed on real patients, after intervention		Significantly better performance in task specific checklist and global assessment score in VR group			Yes
Ahlberg *et al*, 2007 ^29^	Single-blind, randomised control trial; prospective		13 Surgical Residents (all PGY1 & 2)		Training on VR simulator vs no simulation training		Task completion time; error score		Evaluation of operative procedure performed between 5 to 10 times on real patients, after intervention		VR group made significantly fewer errors; task completion time shorter in VR group bit did not reach significance			Yes
Cosman *et al*, 2007 ^30^	Single-blind, randomised control trial; prospective		10 Surgical Trainees		Training on VR simulator vs no simulation training		Task completion time; error assessment score; “bimanual co-ordination”; global assessment score		Evaluation of operative procedure performed on real patient, after intervention		Significant improvement in error score, bi-manual co-ordination and global assessment score in VR group; borderline improvement in task completion time in VR group			Yes
Dawson *et al*, 2007 ^31^	Cohort study; prospective; no evidence of randomisation		9 Surgical Residents		Participation in “skills workshop”, including 8 hours of VR simulation training		5 procedure-related metrics, including task completion time		Evaluation on simulated procedure, before and after intervention		Significant improvement in 3 out of 5 outcome measures, including task completion time, fluoroscopy time and amount of contrast used			No
Balasundaram, Aggarwal & Darzi, 2008 ^32^	Cohort study; prospective; no evidence of randomisation		10 Junior Surgical Residents		Repetition of 5 tasks on VR simulator		Task completion time; error score; instrument path-length		Evaluation over 5 tasks on VR simulator, repeated 10 times each		Significant learning curve seen for task completion time and instrument path-length, but not for error score			No
Verdaasdonk *et al*, 2008 ^33^	Single-blind, randomised control trial; prospective		20 Surgical Trainees (all 1^st^ and 2^nd^ year)		Training on VR simulator vs no simulation training		Task completion time; error assessment score; global rating scale		Evaluation of technical procedure performed on anaesthetised pig, after intervention		Significant improvement in task completion time and error score in VR group; no difference found in global rating scale			No
Larsen *et al*, 2009 ^34^	Single-blind, randomised control trial; prospective		24 Obstetrics & Gynaecology Registrars enrolled, 21 completed (PGY3 to 8)		Training on VR simulator vs “standard” clinical training		Task completion time; performance score		Evaluation of technical procedure performed on real patient, after intervention		Task completion time significantly shorter and performance score significantly higher in VR group			Yes

*PGY = post-graduate year; †T = Trainee on “traditional” training programme; ‡Study by McClusky et al available as abstract only; ¶“Critical error” rate refers to those errors that in real patients would result in potential neurological damage.

### Bench models and box trainers

Nine studies were included that detail the use of bench models and box-trainers; 8 studies were randomised-controlled trials,^13^^-^^19^^,^^21^ with 1 cohort study.^20^ Bench models are simulators that are static, and can be placed on the “bench” in front of the trainee. They use a wide variety of materials that allow the practice of skills such as knot tying, suturing (e.g. wound closure) or anastomoses (the joining of two structures, e.g. re-joining a segment of bowel to bowel, or joining blood vessels together). They are considered low-fidelity, as the materials used can be as simple as pieces of string, beads, metal hooks or stretched elastic bands. Box-trainers are used to simulate laparoscopic (“key-hole”) surgery. The “box” usually has slits in its surface through which surgical instruments, including the laparoscopic camera, can be inserted. The trainee can then use the surgical instruments to manipulate materials placed inside the box. These materials can be as simple as the bench models described above, or of higher fidelity by using animal tissues. This category contained the only study to conclude that the improvement seen in operative skill was independent of simulation training.^17^

### Virtual reality

Fourteen studies meeting the inclusion criteria were found. 11 of these were randomised-controlled trials,^22^^-^^24^^,^^26^^-^^30^^,^^33^^-^^35^ with 3 cohort studies.^25^^,^^31^^,^^33^ Three of the randomised-controlled trials purport to be “double-blinded”,^23^^,^^26^^,^^27^ however, whilst those performing the final evaluation are blinded in each study, it is impossible to blind the participants themselves to which intervention group they are in. Participant blinding would be necessary for it to be considered a double-blinded study. VR simulation describes the interaction between the trainee and a three-dimensional (3D), computer generated environment.

**Table:** 

**Study**	**Methodology**	**Participants**	** Intervention (simulation task)**	**Outcome measures**	**Method of evaluation**	**Results**	**Evidence of transfer to clinical environment?**
**Maschuw ** ***et al*, 2011 ****^35^**	Single-blind, randomised control trial; prospective	50 Surgical Residents (all PGY1)	Training on VR simulator vs no simulator training	Task completion time, “tissue damage” score; “economy of motion” score	Evaluation of 7 tasks performed on VR simulator, before and after intervention	Significant improvement in all outcome measures in VR group	No
**Fried *et al*, 1999 ****^36^**	Randomised control trial; prospective	12 Surgical Residents, all PGY3	5 weekly practice sessions on inanimate box trainer, vs. no practice	Performance score (PS) based on task completion time and precision of performance	7 different operative tasks, evaluated on inanimate simulator and anaesthetized pig model, before and after intervention	PS significantly increased in practice group in 5 out of 7 tasks, vs 1 out of 7 task for no practice group	No
**Bijoy Thomas ** ***et al*, 2010 ****^37^**	Cohort study; prospective; no evidence of randomisation	31 Obstetrics & Gynaecology Residents – 7 PGY1, 8 PGY2, 11 PGY3, 5 PGY4	1 hour practice session under direct supervision, further 1-2 hours practice session unsupervised, using *ex vivo* porcine model	Subjective self-assessment questionnaire using 10-point Likert Scale	Pre and post-intervention self-assessment questionnaire	Significant improvement in self-perception of comfort level and knowledge of procedure, and of familiarity with surgical instruments	No
**Martin *et al,* 1998 ****^38^**	Cohort study; prospective; no evidence of randomisation	8 Surgical Residents, all PGY1^1^	Cadaveric laboratory, practising 3 technical procedures on 2 occasions 3 weeks apart	Task completion time and number of complications	Evaluation of 3 procedures on cadaver, immediately post-instruction & 3 weeks later	Completion time + no. of complications. decreased significantly, both post-instruction + 3 weeks later	Yes – participants evaluated on real patients following completion of cadaveric laboratory training
**Anastakis ** ***et al*, 1999 ****^39^**	Single-blind, randomised control trial; prospective	23 Surgical Residents, all PGY1	Training on human cadaver vs. training on inanimate bench model vs. learning from prepared text only	Task-specific checklist and global rating scale of operative skill	Performance of 6 different operative procedures on human cadaver; no pre-intervention measurement	Checklist & global score significantly higher after bench and cadaver training; results of bench and cadaver training equivalent	No
**Bergeson ** ***et al*, 2008 ****^40^**	Cohort study; prospective; no evidence of randomisation	3 Orthopaedic Residents - 2 PGY1, 1 PGY3	Use of vertebral body from cadaveric thoracic spines	Error rate, “critical” error rate^¶^, error awareness rate	Consecutive instrumentation of vertebral bodies from 5 cadaveric spine	Error rate significantly decreased 3^rd^ to 5^th^ spines, “critical” error rate significantly decreased 4^th^ & 5^th^ spines	No
**Martin *et al*, 2011 ****^41^**	Cohort study; prospective; no evidence of randomisation	15 Orthopaedic Residents + 4 Attending Surgeons	3 repetitions of virtual reality (VR) shoulder arthroscopy programme	Task completion time	Performance of shoulder arthroscopy task on human cadaver; no pre-intervention measurement	Strong correlation between task completion time on VR programme and on cadaver; task completion time on VR programme significant predictor of task completion time on cadaver	No
**Mehrabi ** ***et al*, 2006 ****^42^**	Cohort study; prospective; assessors calculating performance score (PS) blinded to participants name and experience level	4 Surgeons (including 2 trainees) – 1 Intern; 1 Resident; 1 Fellow and 1 Attending Surgeon	16 consecutive operations on rat model using the Da Vinci robotic system	Task completion time, number of complications and global PS	4 operations on anaesthetised pig using the Da Vinci robotic system, performed before and after intervention	Task completion time significantly lower in 3 out of 4 operations post-intervention; median no. of complications significantly lower and PS significantly higher in all 4 operations post-intervention	No
**Moles *et al*, 2009 ****^43^**	Cohort study; prospective; no evidence of randomisation	7 Surgical Residents, all PGY2 to 5	5 technical tasks repeated 3 times on an inanimate model using the Da Vinci robotic system	Task completion time, number of errors, severity of errors; composite performance score (PS) based on the three parameters above (lower the score, the better the performance)	Each task and its repetition evaluated for each participant; no pre-intervention measurements	Mean task completion time decreased from 1^st^ to 2^nd^ and from 2^nd^ to 3^rd^ trial, not significant; mean number of errors decreased from trial to trial, not significant; composite score significantly decreased from trial to trial	No
**Finan *et al*, 2010 ****^44^**	Cohort study; prospective; no evidence of randomisation	16 Obstetrics & Gynaecology Residents, 3 PGY2, 7 PGY3, 4 PGY4	Completion of training course over 12 months, 3-4 hrs each session, completing 5 surgical procedures on an inanimate model using the Da Vinci robotic system	Number of complications	Evaluation of complication rate on transfer to real patients; no pre-intervention measurements	No complications attributable to resident training observed	Yes – 10 out of 16 participants evaluated on part of or whole operative procedure on real patients

This 3D environment is usually displayed on a computer screen, with the trainee interacting via a computer interface consisting of modified surgical instruments. The simulated environment allows the practice of particular technical exercises, component parts of a particular procedure, or the completion of entire operative procedures e.g. laparoscopic cholecystectomy (key-hole excision of the gallbladder). The completion of an entire operation is also known as “procedural simulation”. VR simulators are also able to provide haptic “force feedback”, where the operating surgeon experiences force, motion and vibration through the surgical instruments being used, as if they were actually touching the patient directly themselves.^9^^,^^45^

### Animal models

Two studies met the inclusion criteria that described the use of an animal model in surgical simulation, with one randomised-controlled trial^36^ and 1 cohort study.^37^ They describe two different animal models. The first is the use of animals in vivo (Latin, “within the living”), where part of or an entire operative procedure is performed on the whole, live anaesthetised or freshly-killed animal. This is considered high-fidelity simulation, where although there are differences between animal and human anatomy, the identification and control of intra-operative bleeding, sensitive tissue handling and awareness of spatial relationships all closely mimic the real operative environment.^46^ The second animal model is considered to be lower fidelity than the first, and describes the use of animal tissue ex vivo (Latin, “out of the living”). Here, surgical tasks are simulated on organs or tissue that has been removed completely from the animal, e.g. the use of animal small bowel to practice small bowel anastomosis (the technique of re-joining divided bowel). The practice of surgical simulation procedures in anaesthetised animals in vivo is currently prohibited by law in the United Kingdom, but is permitted in other European countries, as well as the United States and elsewhere.^45^

### Human cadavers

The use of human cadavers (the donation of the human body after death) was described in a total of four studies that met the final inclusion criteria. Only one of these was a randomised-control trial,^39^ the remainder were cohort studies.^38^^,^^40^^,^^41^ Cadaveric simulation provides a high-fidelity model in which the exact anatomical relationships present in live surgical patients are preserved, with almost identical tissue handling and spatial relationships to that of live surgery. Human cadavers can be used in part or in whole, and a single cadaver can provide the opportunity for more than one trainee to perform more than one procedure or task. In the United Kingdom, the practice of operative procedures on human cadavers by surgeons was made possible with the passing of the Human Tissue Act in 2004.^47^

### Robotics

Three studies describing the use of robotic simulation were included, all of which were cohort studies.^42^^-^^44^ Robotic systems in surgery are also known as “telemanipulators”, and consist of a “robotic stack.” This stack interfaces with the patient, and is controlled by the surgeon via an operating console. The stack itself consists of a varying number of robotic “arms” that hold various surgical instruments – thus it is the robot that performs the operative procedure, under the surgeons’ control. Advantages of robotic systems include the ability to project a stable, tremor-free 3-dimensional operative image; use of tremor-free instruments with 7 degrees of freedom of movement and the ability to experience haptic “force feedback”.^48^ Robotic systems are used in surgical simulation to directly improve robotic surgical skills; the robotic system is used by the trainee to perform simulated exercises on either an animal model or an inanimate bench model. The commonest robotic system in clinical use is the da VinciTM robot (Intuitive Surgical, California, USA).

## Discussion

This review demonstrates the benefits of surgical simulation in the development of technical competence. Improvements in outcome measures are demonstrated in every study, across all five main simulation categories. Only one study found such improvements to be independent of simulation training.^17^ These improvements are shown in both the evaluation of technical performance in the simulated environment, and on transfer to the real patient in the operating room. The exception to this is the use of animal models – neither of the two studies included here attempted to demonstrate transfer of simulated skills to the real operating room environment. Where studies compared the use of different simulation techniques, the evidence suggests that the use of bench models and cadaveric simulation is equivalent,^39^ as is the use of bench models and live animals.^36^ Skills learnt on VR and box trainers were also shown to be transferable between the two techniques, with VR simulation providing a greater improvement in the real operating room.^22^

Although an improvement can be seen with the use of each type of simulation technique, several important issues are raised by the various study designs and methodologies, and the variable quality of the research. The first of these is the outcome measures that are used. Over two-thirds of the studies reviewed here use task completion time as a marker of technical competence. Operative speed has been shown to be an objective measurement of technical skill.^49^ However, the time required to complete an operation has many variables, including factors that lie outside of the surgeons’ control (e.g. patient factors such as the severity of the disease process, and variable anatomy). The ability to operate quickly does not always equate to the ability to operate safely, and it has therefore been argued that although it shows some objectivity, operating speed is a crude measure of skill.^50^ Where studies use only operative speed, trainees and trainers should be wary of accepting such evidence as proof of the effectiveness of simulation.The issue of operative safety is addressed in many of the studies, with the use of outcome measures that calculate error scores and complication rates. On transfer to the real patient, it can be hypothesized that a reduction in error scores would lead to an improvement in patient outcome. A recent systematic review of technology-enhanced simulation for health professions learners included a total of 609 studies, but only 32 of these studies reported effects on patient care, with a moderate pooled effect.^51^ This highlights a significant difficulty in simulation research. The ultimate purpose of simulation is to develop and improve skills that will be transferred to the real operating room, with the end result being the safe completion of an operation that has improved the patients’ health. However, the use of patient outcomes as an outcome measure has significant limitations – first, patient outcomes are affected by many variables that lie outside of the surgeons’ control, much like operation speed. Secondly, there is an “ethical imperative” that the supervised trainee performs to the same standard as the supervising “expert” in terms of patient outcomes, regardless of the level of that trainees’ skill or experience – if this were not the case, trainees would not be allowed to operate at all.^34^ This “ethical imperative” will always exist in surgical practice, and those involved in both the use of, and research in to, surgical simulation must be aware of this limitation.Other outcome measures used in several of the studies are the task-specific checklist and the global assessment score. Both of these measures have been shown to be reliable, valid and objective measures of technical skill.^52^^,^^53^ Of the two rating scales, however, it has been suggested that the global assessment score is the more reliable.^54^ The use of task-specific checklists, and other task or procedure specific outcomes also poses difficulties in the generalisation of results – simulation that improves a task specific checklist for a laparoscopic gallbladder operation can be generalised to trainees performing that particular procedure, but could not be generalised to a cardiac surgeon performing a valve repair, or an orthopaedic surgeon performing a hip replacement, as the task checklist would have little relevance. It should also be noted that all of the studies described in the “Robotics” category are in fact task-specific to robotic surgery – these simulation studies all use the robotic stack, with a skill-set that is tailored to robotics. Although these studies demonstrate an improvement in technical competence, their results and conclusions should therefore not be generalised beyond the scope of robotics and further research to identify whether robotic skills are transferable to other arenas is necessary.The transfer of skills between different simulation tools is addressed by a small number of studies, suggesting that skills can be transferred from VR to the human cadaver,^41^ and from the box trainer to VR and vice versa.^22^ In addition, in those studies that attempt to demonstrate transfer of skills from the simulated environment to the real patient (13 studies in total), all but one showed simulation to be effective on transfer. However, it has also been demonstrated elsewhere that specific skill sets in surgery need specific targeted training – Figert et al^55^ showed that surgeons with considerable experience of open surgery but limited laparoscopic experience were not able to transfer their open surgery experience to newly-acquired laparoscopic skills. Many of the studies reviewed here evaluate laparoscopic skills and laparoscopic procedures. Generalising the results of these studies to non-laparoscopic skills and procedures, and across surgical specialties that use little or no laparoscopic techniques should therefore be attempted with caution.The heterogeneity of the outcome measures used has been highlighted in previous work on the quality of surgical simulation research.^56^ The same authors also found that studies in surgical simulation often had small participant numbers and lacked statistical power calculations to support their sample sizes. As well as disparate outcome measures, they also commented on the disparate simulation interventions themselves. The review detailed here supports some of these findings. The largest two studies included 50 participants, but a full 27 studies included less than half of this number, with 7 studies having 10 participants or less – the smallest study size had only 3 participants.^40^A statistical power calculation was only found in 2 studies.^21^^,^^34^ There is also little uniformity in the simulation exercises, the simulation equipment (e.g. different VR systems and software) or the frequency with which the simulation exercises are performed and practiced. Maargard et al^57^ have demonstrated that without continual training, skills learnt on a VR simulator were retained at 6 months, but deteriorated between 6 and 18 months. Therefore the issue of simulation frequency highlights an area for further research, to address whether simulation has a lasting effect or whether skills learnt decay over time, and how often trainees should undergo simulation training in order to keep up their skills.A further feature of the disparate nature of the simulation techniques being reviewed here is the use of “simulation plus…” i.e. the use of simulation as part of a “skills curriculum”, accompanied by structured, mentored feedback and instruction, or used in addition to traditional operating room training. Those studies that describe a “skills curriculum” combine simulation with a mixture of demonstration videos, didactic instruction, lectures, procedural demonstrations by experts and/or written material. However, no attempt is made to separate the effect of the simulation exercises from these additional teaching modalities. Therefore, whilst the benefit of these curricula can clearly be seen, the precise contribution of their individual components is less so. These difficulties are compounded by the fact that no two “skills curriculae” described in these simulation studies are exactly alike.The precise influence of instruction and feedback during simulation practice is also unclear in most of the studies, as once again it is not separated from the simulation exercise itself. The exception is the study by Risucci et al^16^, who specifically set out to determine the effect of simulation practice and additional instruction on laparoscopic skills. This additional instruction takes the form of a demonstration video and tutor feedback. Those undergoing simulation plus instruction showed an improvement in task completion times, plus a greater improvement in both error rate and in variability of performance. This suggests that simulation practice is augmented by instruction and feedback from an expert tutor. The influence of feedback is not unnoticed in the wider educational literature. Feedback is seen as an integral part of Kolb’s experiential learning cycle, where the learners’ ideas are formed and modified through experience.^58^ Hill argues that feedback plays an important role in Kolb’s cycle as it supports the process of reflection and the consideration of new and more in-depth theories, and helps the learner plan more productively for their next learning experience.^59^Improving classroom learning through the use of assessment has also been shown to be dependent on the provision of feedback to learners, in order to help them recognise both the standards they are aiming for and the next steps they need to take in the learning process.^60^ The lack of emphasis on feedback in surgical simulation studies may be because such feedback from expert tutors must come at an additional cost in both man-power and time that is potentially difficult to meet.The additional effect of instruction and feedback also suggests that simple “quantity” and repetition of simulation exercises is not sufficient; rather, it is both the “quantity” and the “quality” of simulation that is important. This is borne out by Joyce et al,^20^ who showed that although simulation improved performance, no correlation was found between the amount of time spent practising and task completion time, with only a low correlation between practice time and technical skill. These findings on “quality vs quantity” are supported by Ericsson et al’s theory of “deliberate practice.” They suggest that simply having sufficient experience or undergoing a sufficient amount of practice is not enough for the achievement of maximal performance; rather, it is the precise nature of the practice itself that leads to maximal performance.They define “deliberate practice” as “activities that have been specifically designed to improve the current level or performance,” and propose that deliberate practice must take into account the learners motivation and their pre-existing knowledge, must be accompanied by immediate formative feedback, and should extend over a period of at least 10 years in order for “expert performance” to be achieved.^61^

### Limitations of the study

The literature on simulation in surgery is international, with a significant amount of work from the US. In order to compare such international research, the notion of a “generic level” of basic clinical competence before embarking on surgical training is therefore an assumption - that surgical trainees across differing countries all achieve the same basic level of competence before their surgical training begins. In evaluating the literature on the use of surgical simulation across different surgical specialties, another assumption is made, that the technical skills and tasks practiced repeatedly in one specialty are generic and transferable between all specialties. A further inherent limitation to the use of a literature-based methodology is that the conclusions of the review rely heavily on the methodological adequacies, or inadequacies, of the studies included; such conclusions must therefore take into account the quality and rigour of the research being reviewed. This review has also been conducted by a single author, who was solely responsible for the selection of the included studies and for data analysis; this introduces the potential for bias.

## Conclusions

- Five main categories of simulation technique currently used to develop technical competence in surgical training are identified here – the use of bench models and box trainers; Virtual Reality; human cadavers; animal models and robotics. On reviewing the available evidence, the benefits of all five of these techniques in improving technical skills can be seen within the simulated environment. All but the use of animal models show the ability to transfer skills to the real patient in the operating room environment. Therefore surgical trainees should be confident in the effects of using simulation during their training, and those involved in the planning of surgical training should endeavour to provide trainees with access to formal, structured simulation.
- When considering the evidence for surgical simulation, both trainees and trainers should be aware of the task-specific nature of surgical simulation research. As a consequence, trainees should tailor their simulation training to those simulation exercises designed to improve the skills that form a significant part of their daily practice (e.g. laparoscopic exercises for those that practice laparoscopic techniques). When designing surgical training, educational leaders must also be aware that surgical simulation needs to be tailored to the needs of individual trainees in individual specialties.
- The use of cadaveric simulation is equivalent to the use of VR or box trainers. Due to the scarcity of cadaveric material, and the ethical and moral issues around its use, resources should be directed towards training on VR and box trainers.
- Only a very small number of studies detail the use of animal models in surgical trainees, and they did not attempt to demonstrate transfer of skills to the real  patient. The use of a bench model was also shown to be equivalent to the use of live animals. Therefore, as animal models also carry significant ethical and moral  issues, and the use of live animals is prohibited in the UK, other methods of simulation should be considered when planning surgical training.
- Skills learnt on both box trainers and VR are transferable to the real patient, with the evidence suggesting the slight superiority of VR. However, VR equipment is more expensive. Surgical skills curricula should therefore incorporate simulation on box trainers, with VR being used in addition, where resources allow.
- Simulation on robotic systems has a direct effect on the development of robotic skills, but whether such skills transfer to other surgical arenas is unknown. With the cost of robotic systems so high, robotic simulation should only be considered in those trainees who will definitely need to use robotics in their daily practice; the number of such trainees is limited at present due to the very small number of centres utilising robotics.
- To enhance the benefits of structured simulation, trainers should provide time for trainees to receive expert instruction and feedback during their simulation training. Such feedback should be delivered in additional to self-directed practice.
- Areas for future research in surgical simulation include the determination of how skills learnt during simulation exercises are retained, the frequency and intensity of simulation that provides the maximum benefit, and further work on the transfer of skills between different simulation techniques, particularly the transfer of  robotic skills. The complex nature of educational interventions must also be recognised by those planning and evaluating surgical simulation research, particularly when designing a “skills curriculum”.

### Conflict of Interest

The author declares that he has no conflict of interest.
